# Factors Related to Parental Intention to Vaccinate Young Adolescent Boys Against Human Papillomavirus in Taiwan

**DOI:** 10.1002/kjm2.70160

**Published:** 2025-12-26

**Authors:** Yu‐Min Chen, Yu‐Ping Chang, Cheng‐Fang Yen

**Affiliations:** ^1^ Department of Psychiatry Kaohsiung Medical University Hospital Kaohsiung Taiwan; ^2^ School of Nursing State University of New York, University at Buffalo New York USA; ^3^ Department of Psychiatry, School of Medicine, College of Medicine Kaohsiung Medical University Kaohsiung Taiwan

Dear Editor,

1

Both men and women carry a 50%–80% lifetime risk of contracting human papillomavirus (HPV) [1]. HPV infection increases the risks of cervical and oral malignancies, spontaneous abortions, and male infertility [1, 2]. Taiwan's Health Promotion Administration has decided to subsidize free HPV vaccination for junior high school boys. However, parents' concerns regarding potential side effects of HPV vaccines may influence their intention to vaccinate their children. This study investigated the determinants of parental intention to vaccinate their sons against HPV in Taiwanese parents.

This study enrolled 220 Taiwanese parents (170 mothers and 50 fathers) who had sons aged between 12 and 15 through online advertisements posted on social media platforms from January 2025 to April 2025. Participants were asked about their intention to vaccinate their sons against HPV by using the following question: “Please rate your current willingness to let your son receive HPV vaccination.” Responses were rated on a Likert scale ranging from 1 (*very low*) to 10 (*very high*). Participants' cognitive motors to vaccinate their sons against HPV was assessed using the Parental Motors of Human Papillomavirus Vaccination Acceptance Scale (P‐MoVac‐HPV‐12) pertaining to the respondent's perceived effect, knowledge, and value of the HPV vaccine and autonomy in decision‐making regarding their child's health care [3]. Participants' understanding of the government's rationale for promoting HPV vaccination, concerns regarding the side effects of HPV vaccines, awareness of reported side effects of HPV vaccination experienced by some individuals, and exposure to information about HPV vaccines from social media were also collected.

The mean score for parental intention to vaccinate their sons against HPV was 7.05 (SD = 2.56). Table 1 presents parents' characteristics and the results on the factors related to parental intention to vaccinate their sons against HPV. In bivariable linear regression analysis models, eight variables were significantly correlated with parental intention to vaccinate their sons. These factors significantly associated with parental intention were subsequently included in a stepwise multivariable linear regression model. The results indicated that perceived value of HPV vaccination, autonomy in deciding, and understanding of the government's rationale were positively associated with parental intention, whereas worry regarding the side effects was negatively associated with parental intention. Mothers had a higher level of intention to vaccinate their sons than fathers.

**TABLE 1:** Factors related to parental intention to vaccinate their sons against HPV: Bivariable and stepwise multivariable linear regression analysis.

	*n* (%) or mean (SD)	Bivariable linear regression		Stepwise multivariable linear regression	
		B (se)	*p*	B (se)	*p*
Sex (0 = female, 1 = male)	50 (22.7)	−0.971 (0.408)	0.018	−0.470 (0.231)	0.043
Education level (0 = high school or below, 1 = college or above)	194 (88.2)	0.667 (0.534)	0.213		
Exposure to information from social media (0 = no, 1 = yes)	127 (57.7)	0.981 (0.193)	< 0.001		
Awareness of side effects of HPV vaccination (0 = no, 1 = yes)	60 (27.3)	−0.229 (0.388)	0.556		
Age (years)	44.36 (5.95)	0.019 (0.029)	0.510		
Perceived effects of HPV vaccination	16.65 (2.88)	0.547 (0.046)	< 0.001	0.411 (0.040)	< 0.001
Knowledge of HPV vaccination	14.38 (3.60)	0.499 (0.034)	< 0.001		
Perceived value of HPV vaccination for health	16.77 (3.02)	0.627 (0.039)	< 0.001		
Autonomy in deciding to vaccinate	13.11 (3.31)	0.327 (0.047)	< 0.001	0.139 (0.031)	< 0.001
Understanding of the government's rationale	1.57 (0.92)	1.407 (0.162)	< 0.001	0.317 (0.122)	0.010
Worry regarding the side effects of HPV vaccines	1.59 (0.68)	−2.505 (0.191)	< 0.001	−1.102 (0.178)	< 0.001
Boys' age (years)	13.49 (0.92)	0.164 (0.187)	0.383		

Abbreviations: HPV: human papillomavirus; SE: standard error.

According to the theory of planned behavior (TPB) [4], the intention to receive vaccinations is shaped by one's attitude toward vaccination, subjective norms of vaccination, and perceived autonomy in deciding whether to receive vaccination. This study supported the concepts of TPM by confirming the associations of perceived effects of HPV vaccination, worry regarding the side effects, and autonomy in deciding with the intention to vaccinate their sons. However, this study did not measure parents' subjective norms of HPV vaccination. Further, according to the protection motivation theory (PMT) [5], the intention to receive vaccinations is influenced by individuals' evaluations of the severity of HPV, vulnerability to HPV, and one's ability to cope with HPV. Although this study did not directly measure perceived severity and vulnerability of HPV, parents may be aware of them through the government's promotion of HPV vaccination. This study recommends integrating these factors into the design of intervention programs aimed at enhancing parental intention to vaccinate their sons against HPV. Further study is needed to examine the associations of parent's subjective norms and appraisal of efficacy to cope with HPV with parental intention.

The sample of this study was recruited via social media advertisements and included a disproportionately high number of mothers. They might limit the generalizability of the results of this study.

## Funding

This work was supported by Kaohsiung Medical University Chung‐Ho Memorial Hospital, KMUH113‐M302.

## Conflicts of Interest

The authors declare no conflicts of interest.

## Data Availability

The data that support the findings of this study are available on request from the corresponding author. The data are not publicly available due to privacy or ethical restrictions.
